# The transcriptional dynamics of *TIFY/JAZ* and *BBX* families in jasmonate signaling reveal SlBBX17 as a positive regulator of tomato defense

**DOI:** 10.1007/s00299-026-03893-8

**Published:** 2026-07-04

**Authors:** Bruno Silvestre Lira, Letícia Guimarães Barbosa, Marcelo Lattarulo Campos, Juliene Moreira, Gabriel Ponciano, Raquel Tsu Ay Wu, Lumi Shiose, Nikolaos Ntelkis, Nathalia de Setta, Alain Goossens, Luciano Freschi, Magdalena Rossi

**Affiliations:** 1 Departamento de Botânica, Instituto de Biociências, Universidade de São Paulo, Rua do Matão 277, São Paulo, 05508-090 Brazil; 2 Departamento de Botânica e Ecologia, Instituto de Biociências, Universidade Federal de Mato Grosso, Av. Fernando Corrêa da Costa 2367, Mato Grosso, 78060-900 Brazil; 3 Department of Plant Biotechnology and Bioinformatics, Ghent University, Technologiepark-Zwijnaarde 71, Ghent, Belgium; 4 Center for Plant Systems Biology, VIB, Technologiepark-Zwijnaarde 71, Ghent, Belgium; 5 Centro de Ciências Naturais e Humanas, Universidade Federal do ABC, Alameda da Universidade S/N, São Bernardo do Campo, 09606-405 Brazil; 6 Department of Botany and Zoology, Stellenbosch University, Stellenbosch, 7600 South Africa

**Keywords:** *Spodoptera frugiperda*, MYC2, Herbivory, Mechanical wounding, B-BOX domain, Biotic stress

## Abstract

**Key message:**

SlBBX17 positively regulates tomato defense by modulating JA-mediated signaling.

**Abstract:**

Jasmonic acid (JA) is a key plant defense metabolite, particularly against herbivory and necrotrophic pathogens. The MYC2 transcription factor (TF), a core TF of the JA response, is repressed by its interaction with JAZ proteins, members of the TIFY/JAZ protein family. Biotic stress stimuli trigger the accumulation of the bioactive JA-Ile conjugate, which binds to the SCF^COI1^-JAZ co-receptor complex, promoting the ubiquitination and subsequent degradation of JAZ proteins, thereby releasing MYC2 to activate downstream target genes. JAZ proteins also regulate other signaling pathways through protein–protein interactions with TFs. However, interactions between JAZs and BBX TFs, emerging regulators of several physiological and developmental processes in plants, remain underexplored. In this study, we revisited the SlTIFY/JAZ family in the tomato (*Solanum lycopersicum*) genome, examining their diversity, domain topology, and conserved motifs. Transcriptional profiling of *SlTIFY/JAZs* and *SlBBXs* in tomato leaves revealed three distinct gene groups based on their response to JA, with some being intensely upregulated, displaying *SlMYC2*-like expression, or downregulated. Further investigation of differentially expressed *SlJAZs* and *SlBBXs* in different genotypes of tomato hairy root cultures underscored the dependency of SlCOI1, and, at least partially, of SlMYC1 and/or SlMYC2 for their JA-mediated transcriptional modulation. The expression patterns pinpointed *SlBBX17* as a SlMYC2-dependent JA-responsive gene. Transactivation assays further indicate that SlMYC2 upregulates *SlBBX17* expression. Functional analyses revealed that SlBBX17 positively contributes to plant defense, because loss-of-function genotypes displayed increased susceptibility to *Spodoptera frugiperda* herbivory, whereas overexpression enhanced resistance. Together, our results point to SlBBX17 as a JA-responsive regulator that modulates defense responses.

**Supplementary Information:**

The online version contains supplementary material available at 10.1007/s00299-026-03893-8.

## Introduction

Jasmonic acid (JA) is a lipid-derived signaling molecule primarily known for regulating reproductive development and stress responses, thereby coordinating defense mechanisms and physiological processes such as growth, senescence, and germination (Wasternack and Feussner 2018). The synthesis of this compound begins in the chloroplasts, proceeds in the peroxisomes, and culminates in the cytosol, where distinct modifications can yield more than 30 derivatives, some active and others inactive. Among these, jasmonoyl-L-isoleucine (JA-Ile) is the endogenous bioactive form essential for JA perception and signaling (Fonseca et al. 2009; Sheard et al. 2010; Yan et al. 2016; Wasternack and Feussner 2018).

In the resting state, when JA-Ile levels are low, JA-responsive genes are repressed through the association of the TOPLESS (TPL; Szemenyei et al. 2008), NOVEL INTERACTORS OF JAZ (NINJA; Pauwels et al. 2010), and JASMONATE-ZIM DOMAIN (JAZ; Chini et al. 2007) complex to the MYELOCYTOMATOSIS ONCOGENE 2 (MYC2; Fernández-Calvo et al. 2011) transcription factor (TF). Once a stress or developmental cue triggers the synthesis and accumulation of JA-Ile, it is translocated to the nucleus, where it binds to CORONATINE INSENSITIVE 1 (COI1; Xie et al. 1998)-JAZ co-receptor, a component of the SKP1, CULLIN, F-BOX PROTEIN E3 UBIQUITIN LIGASE (SCF^COI1^) complex. This interaction facilitates SCF^COI1^ binding to the JAZ proteins in the TPL-NINJA-JAZ complex, leading to polyubiquitination and subsequent degradation of the latter by the 26S proteasome (Howe et al. 2018). Consequently, repression of MYC2 is alleviated, resulting in the upregulation of JA-responsive genes (Song et al. 2022). The activity of MYC2 relies on the physical interaction with the MEDIATOR COMPLEX SUBUNIT 25 (MED25; Chen et al. 2012), which facilitates the interaction between SCF^COI1^ and JAZ proteins, and recruits RNA POLYMERASE II and other proteins, including epigenetic regulation-related proteins, to the promoter of MYC2 target genes (Zhai et al. 2020). Notably, JAZ genes are among those rapidly induced by MYC2 itself (Wasternack and Strnad 2016), indicating a negative feedback loop that modulates the JA pathway and prevents the overactivation of immune responses. This regulatory mechanism underscores the pivotal role of JAZs as repressors of JA signaling. These proteins belong to the TIFY/JAZ family characterized by the presence of the TIFY domain, named after the four most conserved amino acids. This family is further divided into four groups, namely TIFY, JAZ, PEAPOD (PPD), and ZINC-FINGER INFLORESCENCE MERISTEM (ZIM), based on the additional domains found in the proteins (Bai et al. 2011). TIFY proteins, which contain only the TIFY domain, have been less functionally characterized. However, in *Arabidopsis thaliana*, AtTIFY8, the sole non-JAZ TIFY group member, was identified as a transcriptional repressor that negatively regulates leaf senescence (Cuéllar Pérez et al. 2014; Andrade Galan et al. 2023). The JAZ group is defined by the presence of the TIFY and Jas domains (Pauwels and Goossens 2011). The domain topology of the PPD subfamily is composed of the PPD, TIFY and a modified Jas domain, with its members known to repress leaf growth, stomatal density, hypocotyl elongation, and seed size, and regulate leaf curvature (White 2006, 2022; Baekelandt et al. 2018). The ZIM subgroup consists of ZIM and ZIM-LIKE (ZML) proteins that harbor TIFY, Jas, GATA zinc finger, and CONSTANS, CONSTANS-like and TIMING OF CAB1 (CCT) domains. ZIM proteins are involved in various processes, including seed germination, cryptochrome-mediated responses to high irradiance, and hypocotyl and petiole elongation (Shikata et al. 2004; Shaikhali et al. 2012; Vélez-Bermúdez et al. 2015; Xu et al. 2020; Kim et al. 2023).

Except for the GATA domain present in the ZIM group, TIFY/JAZ proteins lack DNA-binding domains; thus, their regulatory activity is primarily mediated through protein–protein interactions (Shikata et al. 2003; Chung and Howe 2009; Chini et al. 2009; Bai et al. 2011). This has been particularly well characterized in the JAZ group, where the genes are transcriptionally induced by JA and their protein function relies heavily on post-translational modifications and protein interactions (Chung et al. 2008; Xu et al. 2024). Over the years, the JAZ protein interaction network has been progressively unraveled (Guo et al. 2018; Liu and Timko 2021; Ghorbel et al. 2021; Zhou et al. 2023). Despite this progress, interactions between JAZ proteins and several TF families remain underexplored, limiting our understanding of how JA signaling is fine-tuned in response to different stimuli. In this context, growing evidence suggests that members of the B-BOX (BBX) domain-containing TF family are promising interactors within several JA regulatory hubs (Lira et al. 2026). For instance, AtBBX1, also known as CONSTANS, interacts with six AtJAZs, antagonizing JA signaling in *A. thaliana* seedlings. Surprisingly, the interaction with AtJAZ1 attenuates the repression exerted by AtBBX1 over JA-responsive genes (Han et al. 2023). In contrast, in petal senescence, AtBBX1 promotes JA signaling through its interaction with and inactivation of AtJAZ3 (Serrano-Bueno et al. 2022). In the context of shade avoidance, AtBBX24 interacts synergistically with AtJAZ3 to promote hypocotyl elongation (Saura-Sánchez et al. 2023). In sweet potato (*Ipomoea batatas*), IbBBX24 has been identified as a positive regulator of JA signaling. It not only transcriptionally represses *IbJAZ10* and activates *IbMYC2*, but also physically interacts with IbJAZ10, preventing it from binding to and repressing IbMYC2 activity (Zhang et al. 2020). Finally, in apple (*Malus domestica*), MdJAZ1 and MdJAZ2 bind to MdBBX37, inhibiting its transcriptional activity. Upon cold- or senescence-induced JA accumulation, the degradation of these JAZ proteins enables MdBBX37 to activate the expression of its downstream targets, enhancing cold tolerance and inducing leaf senescence, respectively (An et al. 2021, 2022).

Recently, two tomato (*Solanum lycopersicum*) BBX proteins, namely SlBBX20 (Solyc12g089240) and SlBBX25 (Solyc01g110180), were identified as positive and negative regulators of resistance to the fungus *Botrytis cinerea*, respectively (Luo et al. 2023; Shiose et al. 2024). The response to the necrotrophic *B. cinerea* and to herbivorous insects is mediated by JA (Pierik and Ballaré 2021). To determine whether BBX proteins and TIFY/JAZ factors are functionally interconnected in JA-mediated responses, we revisited the diversity of TIFY/JAZ proteins encoded by the tomato genome and analyzed the transcriptional dynamics of SlBBX and SlTIFY/JAZ genes following JA treatment or mechanical wounding. In addition, functional characterization of *SlBBX17*-deficient and *SlBBX17*-overexpressing plants provided further insight into the regulatory role of BBX proteins in JA signaling.

## Materials and methods

### TIFY/JAZ protein family identification and phylogenetic and evolutionary analyses

Sequences of *A. thaliana* TIFY/JAZ family members were retrieved from The Arabidopsis Information Resource (https://www.arabidopsis.org) and used as a query in Phytozome 13 (https://phytozome-next.jgi.doe.gov/; Goodstein et al., 2012) database to identify protein homologs in the *S. lycopersicum* genome v2.4 and v3.2. The Structural Group I of BBX proteins from these species was used as an outgroup for the analysis (Lira et al. 2020). The sequences (Supplementary Table S1) were aligned with the homology extension T-Coffee algorithm (PSI-Coffee; Notredame et al. 2000). From the obtained alignment, the phylogenetic tree was reconstructed with the PHYML 3.0 (Guindon et al. 2010) package, using the LG substitution model, with the proportion of invariable sites and the gamma shape parameter estimated from the data. The tree topology and branch length were optimized using the subtree pruning and regrafting method, and the branch support was calculated using an SH-like approximate likelihood ratio test.

The domain topology of all identified proteins was predicted using the InterPro database (Blum et al. 2025). For domain consensus analysis, each group was independently aligned as described above, excluding SlJAZ12 (Solyc01g009740) and AtJAZ11 (AT3G43440) due to their abnormal protein size and repeated domains. The logo representation of the identified domains was constructed using WebLogo3 (https://weblogo.threeplusone.com/create.cgi; Crooks et al. 2004). To calculate the evolutionary distance of the amino acid sequences, the consensus was created from the alignments using the consensus maker tool (https://www.hiv.lanl.gov/content/sequence/CONSENSUS/SimpCon.html). The pairwise distances were subsequently estimated via MEGA v12 software (Kumar et al. 2024) using the Poisson substitution model with uniform rates across sites and the pairwise deletion method for gaps/missing data.

For the phylogenetic analysis of tomato BBX family, the protein sequence encoded by the 31 *SlBBX* loci (Supplementary Table S1, Lira et al. 2020) was retrieved from the *S. lycopersicum* genome v2.4. The alignment and tree reconstruction were carried out as described above.

For the evolutionary analysis, JAZ sequences from *S. pennellii* and *S. tuberosum* were retrieved from Sol Genomics Network (https://solgenomics.net/; Fernandez-Pozo et al. 2015) and aligned with *S. lycopersicum* sequences as described above. The sequences used are listed in Supplementary Table S1. To analyze the selective constraints shaping the evolution of JAZ proteins, we performed site and branch-site model tests using the CODEML program from the PAML package (Yang 2007). The site model test evaluates which of the three models fits the diversification of coding sequences best. Model M0 assumes that all codons evolve under a single ω ratio indicative of purifying selection (0 < ω < 1). Model M1a introduces two codon classes: one under purifying selection and another under neutral evolution (ω = 1). Model M2a expands this by incorporating a third class of codons evolving under positive selection (ω > 1). Model fits (M0 vs. M1a, and M1a vs. M2a) were compared using likelihood ratio tests (LRTs), which evaluate twice the difference in log-likelihoods against a Chi-square (Χ^2^) distribution (Yang 2007). The branch-site model was employed to detect positive selection affecting specific codons along pre-specified lineages (Yang and Nielsen 2002). Lineages under test are designated as foreground branches, while the remaining lineages are considered background branches. In the background branches, codons are categorized as either under purifying selection or neutral evolution. In contrast, foreground branches allow for the possibility that some codons are under positive selection. The alternative hypothesis (H1), allowing ω2 > 1 in foreground branches, was compared with the null hypothesis (H0), in which ω2 is fixed at 1 (Yang et al. 2000; Zhang et al. 2005). As with the site test, model fits (H1 vs. H0) were evaluated using LRTs with a Χ^2^ distribution.

### Plant material

Tomato (*S. lycopersicum* cv Micro-Tom) plants were grown in 0.5-L pots containing a 1:1 mixture of a commercial substrate (Topstrato HT, Vida Verde, Brazil) and vermiculite supplemented with 1 g L^−1^ of NPK 10:10:10, 4 g L^−1^ of dolomite limestone, and 2 g L^−1^ Yoorin Master® (Yoorin Fertilizantes, Brazil). Cultivation was carried out in a growth chamber with controlled temperature (25 ± 2 °C day and 20 ± 2 °C night), a 12-h light/12-h dark photoperiod, an incident photoirradiance of approximately 200 μmol m^−2^ s^−1^, and a red to red/far ratio of = 1. Irrigation was performed manually by capillarity.

For JA treatment, 40-day-old tomato plants were transferred to 80-L translucent plastic containers with 10 L of water without (control) or with 50 µM JA (pure liquid, Sigma-Aldrich #392,707) to a gaseous final concentration of 6.25 µM. The containers were sealed and kept in the same conditions as described above. The treatment began at 8 am, and the fourth leaf was collected 4 and 24 h after the start of the treatment. All samples were frozen in liquid N_2_, powdered, and stored at -80 °C until analysis.

For the mechanical wounding experiment, a single transversal injury was applied to the mid-portion of the terminal leaflet from the third and fourth fully expanded leaves of 40-day-old tomato plants using a forceps (Cunha et al. 2023). The leaves were injured at 8 am (2 h into the light period) and were collected 1 and 8 h after treatment. All samples were frozen in liquid N_2_, powdered, and stored at -80 °C until analysis.

### RNA extraction and real-time quantitative PCR (RT-qPCR) analysis

Total RNA was extracted from 100 mg fresh weight (FW) of each sample with the ReliaPrep™ RNA Miniprep Systems (Promega) following the manufacturer’s recommendations. RNA was quantified by spectrophotometric measurement, and its integrity was assessed by agarose gel electrophoresis. RNA samples were treated with DNase I (Invitrogen) for 1 h and then, the cDNA was obtained with the SuperScript™ IV Reverse Transcriptase (Invitrogen) kit following the manufacturer’s recommendations. The qPCR reactions were carried out in a QuantStudio 6 Flex Real-Time PCR system (Applied Biosystems) using 1X GoTaq^®^ qPCR Master Mix (Promega), 200 nM of each primer (Supplementary Table S2), and 2 µL of 1:10 cDNA dilution in a 10-µL final volume. Cycle quantitation (Cq) and PCR efficiency values were obtained from the fluorescence data analyzed with the LinRegPCR software package (Ruijter et al. 2009). Expression values were normalized against the reference gene *SlEXPRESSED* (Quadrana et al. 2013). A permutation test lacking sample distribution assumption (Pfaffl et al. 2002) was used to identify statistically significant differences (*P* < 0.05) in transcript ratios using the algorithms in the fgStatistics software package (Di Rienzo 2009).

### Treatment of tomato hairy roots with JA

Tomato hairy root lines were transformed with a *β-GLUCURONIDASE* (*GUS*) overexpression construct (control), a CRISPR/Cas9 construct targeting *SlCOI1* (*Slcoi1;* Solyc05g052620), or both *SlMYC1* (Solyc08g005050) and *SlMYC2* (Solyc08g076930) (*Slmyc1/myc2*) as described in Swinnen et al. (2022). Tomato hairy roots were grown in 5 mL liquid MS + medium for 2 weeks at 25 °C and 150 rpm, in the dark. After 2 weeks, hairy roots were treated with 50 µM JA for 4 and 24 h, and subsequently harvested and stored at -80 °C until analysis.

### RNA-sequencing (RNA-seq) of tomato hairy roots

RNA was extracted from tomato hairy roots with the RNeasy Plant Mini Kit (Qiagen). Of total RNA, 1–3 µg was sent to the VIB Nucleomics Core for sequencing with the Illumina NovaSeq6000 (20 M; 100 nt single-end reads). Library preparation was done using the TruSeq stranded RNA Sample Preparation Kit v2 (Illumina).

Transcript abundances were generated by mapping the reads on the tomato Exome ITAG4.0 using the Salmon mapping tool. RNA-seq count data were processed and analyzed using DESeq2 (version 1.44; Love et al. 2014) in R. Sample metadata, including genotype, treatment, and timepoint, were incorporated into a DESeqDataSet object with a full factorial design: genotype x treatment x timepoint. Genes with fewer than 10 counts in at least three samples were filtered out.

Differential expression analysis was performed using the Wald test implemented in DESeq2. To investigate the treatment effect (JA *vs.* control) for each genotype and timepoint, contrasts were constructed based on model coefficients reflecting main effects and interactions. Specifically, for each genotype–timepoint combination, relevant main effects and interaction terms were summed to estimate the treatment effect at that condition, applying Benjamini–Hochberg multiple testing correction, and a log_2_ fold change threshold of |1|. Variance stabilizing transformation (VST) was applied for downstream visualization and normalized count extraction.

Raw data and normalized counts are available at Arrayexpress Project code: E-MTAB-14610.

### Subcellular localization

The subcellular localization and putative nuclear localization signal of SlBBX17 were predicted using the cNLS Mapper tool (https://nls-mapper.iab.keio.ac.jp, Kosugi et al. 2009). The coding sequence, without stop codon, of *SlBBX17* was amplified using specific primers (Supplementary Table S2) and cloned into the pCR™ 8/GW/TOPO TA Cloning (Invitrogen) entry vector following the manufacturer’s recommendations. Then, the *SlBBX17* coding sequence was recombined into the pK7FWG2 (Karimi et al. 2002) binary vector using LR clonase II enzyme mix (Invitrogen) following the manufacturer’s recommendations. The *pK7FWG2:SlBBX17* vector was introduced in the *Agrobacterium tumefaciens* strain GV3101. Cultures were resuspended in the infiltration buffer (10 mM MES pH 5.6, 10 mM MgCl_2_, 200 µM acetosyringone (Sigma-Aldrich)) to a final OD_600_ of approximately 0.8, incubated for 4 h in darkness at room temperature, and infiltrated into leaves of 4-week-old *Nicotiana benthamiana* plants. The nuclear marker DAPI (10 µg mL^−1^ water-dissolved; Life Technologies) was infiltrated 30 min before the confocal microscope observation. Confocal analyses were carried out in a Leica TCS SP8 STED 3X confocal system coupled to a Leica DMi 8 microscope (CAIMi IB-USP). The GREEN FLUORESCENT PROTEIN (GFP) signal was captured over a 508–553 nm range after excitation at 488 nm, while DAPI fluorescence was captured over a 415–501 nm range after excitation at 405 nm.

### Generation of *SlBBX17*-deficient and -overexpressing genotypes

To generate plants with impaired SlBBX17 activity, we used CRISPR/Cas9-mediated editing. For the selection of the guide RNA (gRNA) sequence targeting *SlBBX17* (Solyc07g052620), the locus was surveyed for potential PAM sequences with guides featuring high on-target efficiency, low off-target efficiency and adequate secondary structure using CRISPR-P v2.0 suit (Liu et al. 2017). The selected gRNA was cloned into the pDIRECT 22_C binary vector (Čermák et al. 2017) using the primers listed in Supplementary Table S2.

To generate *SlBBX17*-overexpressing plants, the full-length coding sequence was amplified from genomic DNA using the primers listed in Supplementary Table S2, and cloned into the binary vector pK7WG2D,1 (Karimi et al. 2002) under the control of the cauliflower mosaic virus *35S* promoter (CaMV 35S), to obtain the *35S::SlBBX17* construct (Supplementary Figure S2A).

The assembled *SlBBX17-*pDIRECT 22_C and *SlBBX17*-pK7WG2D,1 constructs were transformed into the *A. tumefaciens* GV3101 strain and used to stably transform tomato cotyledon explants according to Pagliuso et al. (2025).

The CRISPR/Cas9-mediated editing was assessed by PCR and sequencing with primers flanking the selected PAM sequences (Supplementary Table S2). Two homozygous *Slbbx17*-knockout genotypes, one harboring an allele with a 5-bp deletion and another with a 7-bp deletion in the coding sequence, were obtained (hereafter referred to as *Slbbx17-5* and *Slbbx17-7*, respectively). Both mutations introduce a premature stop codon, leading to the production of a truncated protein lacking any known functional domain (Supplementary Figures S1A and S1B). The T-DNA was segregated from homozygously edited *Slbbx17* plants in the T_2_ generation. The expression of the mutated alleles was reduced to approximately 30% and 60% of the mRNA levels observed in wild-type (WT) plants for *Slbbx17-5* and *Slbbx17-7*, respectively (Supplementary Figure S1C).

To obtain *SlBBX17*-overexpressing lines, the presence of the T-DNA was first confirmed by PCR amplification of the NPTII resistance gene, followed by analysis of *SlBBX17* expression by qRT-PCR using primers listed in Supplementary Table S2. Two transgenic lines, hereafter referred to as *35S::SlBBX17*-*L1* and *35S::SlBBX17-L2*, which exhibited an up to 40- and 100-fold higher expression level than WT plants, were selected for the experiments (Supplementary Figure S2B). All the experiments were carried out with T_2_ generation plants homozygous for the transgene.

### Herbivory resistance assay

For the herbivory assay, five neonate *Spodoptera frugiperda* larvae (purchased at https://pragas.com.vc/) were reared on top of 10 leaves excised from the middle section of 40-day-old tomato plants. Leaves were kept inside a transparent plastic box with watered cotton on the base to maintain leaf moisture, under controlled conditions (28 °C, 16-h light photoperiod, 250 µmol m^−2^ s^−1^ light intensity). The experiment was conducted for 5 days; then, larvae were removed from the leaves and weighed.

### Yeast-2-hybrid protein–protein interaction

Yeast-2-hybrid assays were carried out as described by Moreira et al. (2025). Briefly, the complete coding sequences of the assayed genes (primers listed in Supplementary Table S2) were cloned either into the pGADT7 Gateway™ vector at the N-terminus of the activation domain (AD) of the GALACTOSIDASE 4 (GAL4) protein or into the pGBT9 Gateway™ vector at the N-terminus of the GAL4 DNA-binding domain (BD) (Cuéllar et al. 2013). For each assayed interaction, the pair of vectors was used to co-transform *Saccharomyces cerevisiae* PJ69-4A strain (James et al. 1996) by the polyethylene glycol (PEG)/lithium acetate method. Transformants were selected on SD (synthetic defined) medium (Takara Bio, Shigo, Japan) lacking leucine and tryptophan. *SlJAZ* cloned into pGBT9 was obtained from Alain Goossens Lab. When necessary, 3-aminotriazole (3AT) was used for autoactivation inhibition.

### Transient expression assay (TEA) in bright yellow-2 (BY-2) cells

The full-length coding sequences of *SlBBX17*, *SlMYC2*, and *SlMED25* were amplified with the primers listed in Supplementary Table S2 and cloned into the p2GW7 destination vector (Vanden-Bossche et al. 2013). The promoter regions of *SlBBX17* (1,582 bp) and *SlMYC2* (1,567 bp) were delimited after a search for putative MYC2 (G-Box CACGTG; PBE-Box CATGTG, López-Vidriero et al. 2021) and BBX (CAC[AGT]TG, G[AT]GAGAGA, GAAA[AG][AT]GA, Li et al. 2024; TGTGNNNATG, Tiwari et al. 2010) binding motifs, respectively, using the PlantPAN 4.0 (https://plantpan.itps.ncku.edu.tw/plantpan4/index.html) platform. The promoter regions were amplified with the primers listed in Supplementary Table S2 and cloned into the pGWL7 destination vector (Vanden-Bossche et al. 2013) harboring the *FIREFLY LUCIFERASE* (*fLUC*) reporter gene.

TEAs were performed in *N. tabacum* BY-2 protoplasts as described by Shiose et al. (2024). For normalization, a vector containing the *RENILLA LUCIFERASE* (*rLUC*) reporter gene under the control of the *CaMV 35S* promoter was used.

### Data analyses

Unless specifically described in the sections above, statistical analyses were conducted using Infostat software (release 29/09/2020; Di Rienzo et al. 2011). The samples were tested for homoscedasticity by Shapiro–Wilk test. If homoscedasticity was observed, samples were compared following a Student’s *t*-test (*P* < 0.05), otherwise, the non-parametric Kruskal–Wallis test (*P* < 0.05) was carried out.

## Results

### TIFY/JAZ proteins cluster in four clades

The tomato TIFY/JAZ protein family was first identified by Chini et al. (2017). To reassess the evolutionary relationship within this family, we employed accurate phylogenetic analysis methods with branch support evaluation (Notredame et al. 2000; Guindon et al. 2010), First, TIFY/JAZ proteins encoded in the tomato genome were identified by querying *A. thaliana* TIFY/JAZ proteins against the Phytozome v13 database (Goodstein et al. 2012). The corresponding tomato sequences were retrieved and screened to exclude those lacking the TIFY domain. As a result, 19 sequences containing the TIFY domain (PF06200), along with the Jas (PF09425), PPD (White 2006), GATA (PF00320), and/or CCT (PF06203) domains, were selected for further analysis. These identified proteins matched those previously reported as members of the tomato TIFY/JAZ family (Chini et al. 2017).

**Fig. 1:**
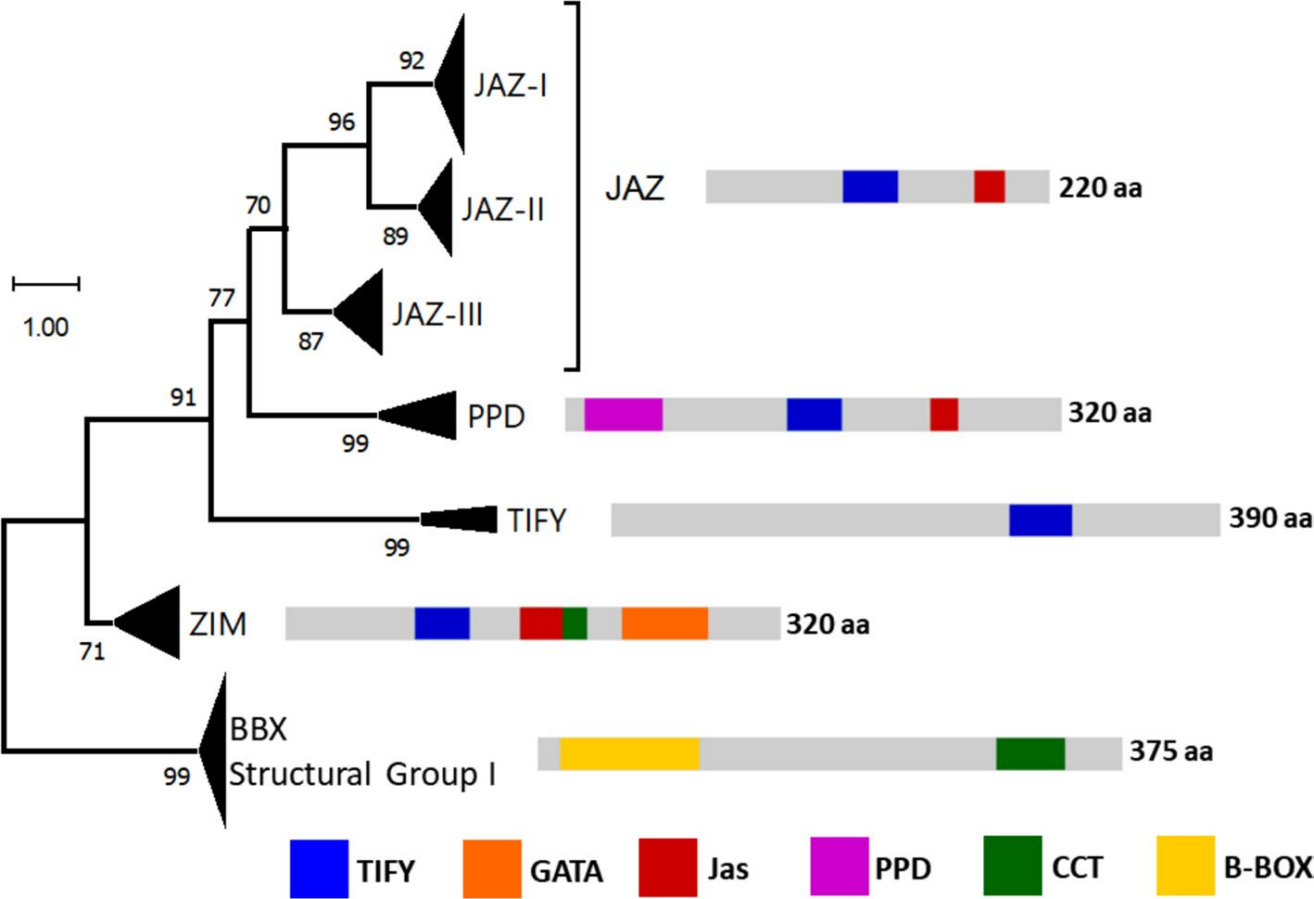
Phylogenetic analysis of *A. thaliana* and *S. lycopersicum* TIFY/JAZ proteins. Condensed representation of the phylogenetic reconstruction obtained from the alignment of *A. thaliana* and *S. lycopersicum* TIFY/JAZ proteins. Clusters were named according to the protein domain signature for each group and the corresponding size is indicated. The domain architecture of each clade was determined by analyzing its consensus sequence. The CCT-containing BBX sequences were used as outgroup for this analysis. The expanded clades are shown in Supplementary Figure S3. Sequences used in this reconstruction are listed in Supplementary Table S1. aa, amino acids

Tomato and *A. thaliana* TIFY/JAZ proteins were aligned by homology extension, along with the CCT-containing Structural Group I of BBX proteins (Lira et al. 2020), which served as outgroup. The phylogenetic tree, reconstructed from the alignment using the maximum likelihood method, revealed four major clades within the TIFY/JAZ family, namely JAZ, PPD, TIFY and ZIM (Fig. 1; Supplementary Figure S3).

The JAZ clade, comprising the shortest sequences with an average length of 220 amino acids, was characterized by the presence of both the TIFY and Jas domains. This clade was clearly subdivided into three subgroups: JAZ-I, JAZ-II, and JAZ-III. The PPD group included proteins averaging 320 amino acids in length, featuring the TIFY, PPD, along with a modified Jas domain. The TIFY proteins, with an average length of approximately 390 amino acids, contained only the TIFY domain. Finally, the ZIM cluster, which branched basally in the phylogeny, comprised proteins averaging 320 amino acids, characterized not only by the previously identified TIFY, GATA zinc finger, and CCT domains (Bai et al. 2011), but also by the presence of a Jas-like domain.

Next, we analyzed the conservation of the domains within each clade. The TIFY domain and its core TIF[F/Y]XG motif (Bai et al. 2011) were identified in all groups. Interestingly, when each group was examined individually, slight variations were observed in the core motif. In JAZ-I, JAZ-II, and JAZ-III groups, the sequences TI[F/I][Y/F]XG, [T/S][I/M]F[Y/F]XG, and T[I/M]FYXG were identified, respectively. In contrast, the canonical TIFYXG motif was preserved in both the PPD and TIFY proteins. As previously reported, the ZIM clade contains the alternative TLS[F/Y]xG consensus for the TIFY domain (Bai et al. 2011), which in our analysis was found as T[L/I]SFXG (Supplementary Figure S4). Notably, the TIFY domains of JAZ-II and JAZ-III subgroups were more similar to each other (0.47) than to JAZ-I, which showed amino acid distances of 1.02 and 1.21 with JAZ-II and JAZ-III, respectively, despite the closer phylogenetic relationship between JAZ-I and JAZ-II clades.

The Jas domain is known to have three conserved regions: the degron ([V/L]P[Q/I]AR[R/K]), responsible for the formation of the COI1/JA-Ile/JAZ complex; the Jas motif (XSLXRFLXKRKXR), which mediates the interactions with other TFs; and the PY-type nuclear localization signal (PY-NLS; X_5_PY) (Bai et al. 2011; Garrido-Bigotes et al. 2019). Here, we identified the degron sequence across all JAZ subgroups, with slight variations from the previously described consensus. The subgroup-specific degron motifs were as follows: [Q/L/P/V][P/K][I/Q/A][A/T/S][R/V/M][K/R] for JAZ-I, [L/E][P/R]IARR for JAZ-II, and [V/L][P/A][Q/L/M][A/T]R[K/R] for JAZ-III. In the corresponding alignment regions of the PPD and ZIM groups, although more divergent, some conserved residues were detectable, displaying the sequences [QHP][AV][NS]RK and x[PS][QEP]R[ALM], respectively (Supplementary Figure S5). The Jas motif appeared as the most conserved region across the five groups. The identified sequences were [K/R/H]SL[Q/H/K][R/S/G]F[L/F][Q/E]KRXXR in JAZ-I, [A/S/N]SL[H/T/Y]RF[L/F][E/A]KRKDR in JAZ-II, A[S/T]LARFLE[K/R]RK[E/H]R in JAZ-III, [A/V][S/L][L/V][Q/E]RY[L/R]EKRK[D/E]R in PPD, and [A/Q]SLXRFR[E/K]KR[K/N]XR in ZIM (Supplementary Figure S5). Finally, the PY-NLS was present without divergence from the described consensus in the JAZ clades, absent in PPD proteins, and reduced to only the conserved terminal tyrosine (Y) in ZIM proteins (Supplementary Figure S5).

Although the amino acid distances across the full-length Jas domain were relatively similar among the JAZ subgroups (ranging from 0.55 to 0.59), analysis of the Jas motif, the largest region within the domain, again revealed a closer relationship between JAZ-II and JAZ-III (0.19) compared to the distances between JAZ-I and JAZ-II (0.45) and between JAZ-I and JAZ-III (0.49). Interestingly, the divergence of JAZ-I from the JAZ-II and JAZ-III groups agrees with the presence of singular structural and/or functional features within the members of this clade. For instance, SlJAZ12 contains six TIFY and two Jas domains; AtJAZ10 possesses an N-terminal cryptic MYC2-interacting domain (CMID, Goossens et al. 2015; Takaoka et al. 2022); AtJAZ8 features a non-canonical Jas domain (Shyu et al. 2012); AtJAZ13 lacks the TIFY domain (Thireault et al. 2015); and SlJAZ9, SlJAZ10, and SlJAZ11 do not bind the SlCOI1-JA-Ile complex (Saito et al. 2021).

To further investigate the diversification of JAZ groups, we assessed whether they have evolved under differential selective constraints. We retrieved the coding sequences of JAZ proteins from the genomes of *Solanum tuberosum* and the wild tomato *Solanum pennellii* and aligned them with the corresponding sequences from *S. lycopersicum*. The reconstructed phylogenetic tree recapitulated the division of JAZ sequences into the three previously identified subgroups (Supplementary Figure S6). We first performed the site model test to evaluate the selective pressures acting on each subgroup. The results indicated that all subgroups are evolving under relaxed purifying selection, with 31% to 57% of codons undergoing neutral evolution (Supplementary Table S3). Next, we applied the branch-site test to evaluate whether specific JAZ loci within each subgroup were subject to positive selection. This analysis identified positively selected codons in *SlJAZ9* and *SlJAZ11*, both belonging to the JAZ-I subgroup (Supplementary Table S3). These findings provided further evidence of structural and/or functional divergence among JAZ proteins and suggested that the subgroups are not evolving under strong purifying selection. Particularly, the JAZ-I subgroup appeared to harbor members undergoing positive selection, supporting its potential role in adaptive diversification.

### SlBBXs and SlTIFY/JAZs expression is modulated by JA

**Fig. 2:**
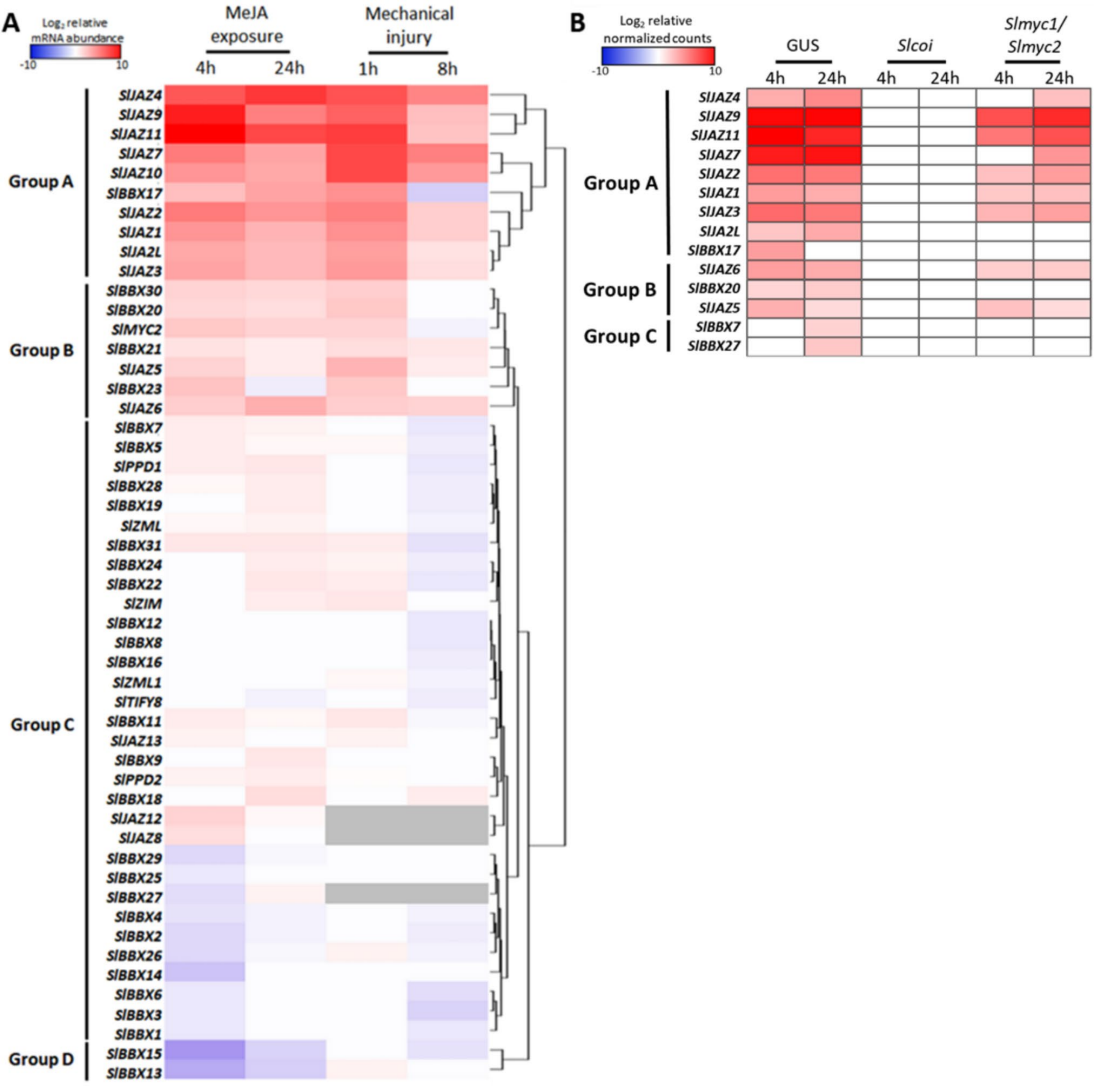
Transcriptional profile of *SlJAZ*s and *SlBBX*s reveals SlCOI1- and SlMYC-dependent responses to JA. **A** Heatmap representation of the transcriptional profile of *SlJAZ*s and *SlBBX*s in leaves exposed to MeJA or mechanically wounded. *SlMYC2* and *SlJA2L* were used as JA-responsive markers. Values were normalized against the respective control sample. Samples were clustered by the average linkage and Euclidean distance method. The heatmap representation was constructed using the Heatmapper web application (Babicki et al. 2016). **B** Heatmap representation of the differentially expressed *SlJAZ*s and *SlBBX*s genes in JA-treated hairy roots of GUS-transformed (control), *Slcoi*, and *Slmyc1/Slmyc2* genotypes. *SlMYC2* and *SlJA2L* were used as JA-response markers. Values are the ratio between the normalized counts of JA-treated and the respective control sample. **A and B** Values represent means of at least three biological replicates. Statistically significant differences relative to the corresponding control sample are colored (adjusted *P* < 0.05). The grey color indicates that expression was not detected. The relative transcript values are detailed in Supplementary Table S4 and S5

The JA response, once triggered by stimuli such as necrotrophic pathogen infection or herbivory (Pierik and Ballaré 2021), is finely tuned by a negative feedback loop in which MYC2 activates the transcription of some *JAZ* genes (Chico et al. 2008). The accumulation of *JAZ* transcripts serves as a molecular marker of JA signaling activity (Pauwels et al. 2009). Moreover, growing evidence pinpoints several BBX proteins as part of the JA signal transduction pathway (Lira et al. 2026). Thus, to determine whether these factors interact in the JA-mediated response and to identify the BBX proteins involved in JA signaling, we analyzed the transcript profiles of *SlTIFY/JAZ* and *SlBBX* in leaves at two timepoints following either sustained exposure to methyl-JA (MeJA) or mechanical wounding to simulate herbivory. Additionally, two JA signaling marker genes were included in the expression analysis: *SlMYC2* and *JASMONIC ACID-2 LIKE* (*SlJA2L*), which is both a regulator of wound-responsive genes and a direct target of SlMYC2 (Du et al. 2017). Interestingly, this analysis revealed four distinct transcriptional patterns (Fig. 2A, Supplementary Table S4). Group A encompassed eight *SlJAZs*, *SlBBX17*, and *SlJA2L*, all of which showed a strong upregulation in response to both treatments, especially at early time points. Group B included *SlMYC2*, four *SlBBX*s, *SlJAZ5*, and *SlJAZ6*, whose transcripts were induced to a lesser extent. Group C consisted of 23 *SlBBX*s, *SlJAZ8*, *SlJAZ12*, *SlJAZ13*, *SlPPD*s, *SlTIFY8*, *SlZIM*, and *SlZML*s that displayed only subtle changes in transcript levels, either up- or downregulated. Finally, Group D encompassed *SlBBX13* and *SlBBX15*, which were strongly downregulated, particularly after MeJA exposure. The expression of *SlBBX10* was not detected in any of the analyzed samples.

Then, we investigated whether JA responsiveness of the surveyed genes depends on COI1 and/or MYC2 by performing RNA-seq on JA-treated *Slcoi1* and *Slmyc1/Slmyc2* mutant hairy root cultures. Of the 51 genes analyzed, 14 were identified as differentially expressed genes (DEGs) between JA-treated and control samples in the control genotype, encompassing *SlJA2L*, nine *SlJAZ*s, and four *SlBBX*s (Fig. 2B, Supplementary Table S5). The absence of these DEGs in *Slcoi1* mutant roots indicates that their JA-mediated upregulation is SlCOI1-dependent. It is worth noting that the expression of *SlBBX17*, *SlBBX20*, *SlBBX7*, *SlBBX27*, and *SlJA2L* was dependent on SlMYC1 and/or SlMYC2, while *SlJAZ*s were only partially modulated by these TFs (Fig. 2B, Supplementary Table S5).

Collectively, analysis of the JA-induced transcriptional response of *SlJAZ*s and *SlBBX*s in leaves revealed four distinct groups: the strongly upregulated group A, the weakly induced group B, and the repressed groups C and D. Additionally, we determined that, with the exception of *SlJAZ10*, the JA-induced transcriptional responsiveness of all group A genes, as well as of *SlJAZ6*, *SlBBX20*, *SlJAZ5*, *SlBBX7*, and *SlBBX27* was COI1-dependent and, at least in part, requires SlMYC1 and/or SlMYC2 activity.

### SlBBX17 regulates plant defense

**Fig. 3:**
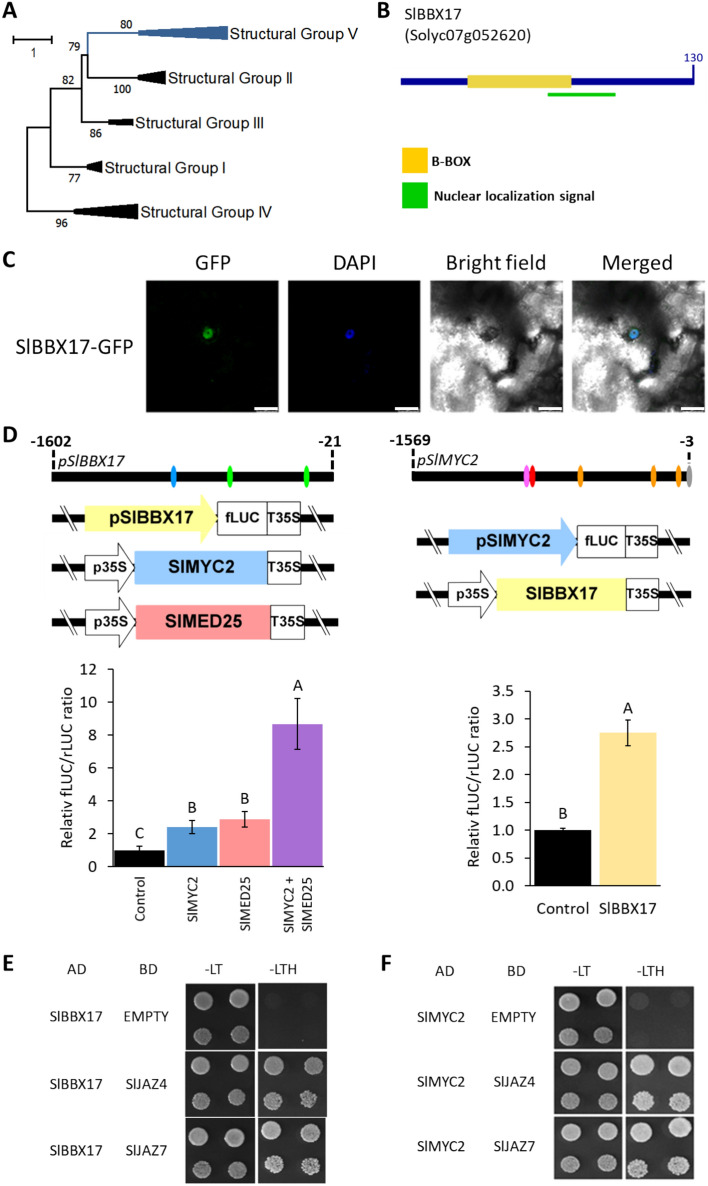
SlBBX17 is a Structural Group V BBX that participates in the JA signal transduction pathway. **A** Phylogenetic reconstruction obtained from the alignment of tomato *(S. lycopersicum*) SlBBXs. Clusters were named following the domain topology described by Lira et al. (2026). SlBBX17 is in the Structural Group V (highlighted in blue). The expanded clades are shown in Supplementary Figure S7. Sequences used in this reconstruction are listed in Supplementary Table S1. **B** Schematic representation of the SlBBX17 protein. Blue lines indicate the regions without known domain; the number indicates the protein length. **C** Subcellular localization of the fusion protein SlBBX17::GFP. Proteins were transiently expressed in *N. benthamiana* leaves by infiltration with *A. tumefaciens*. GFP, DAPI nuclear marker, bright-field and merged signals are indicated above the panels. Scale bars = 25 μm. **D** Putative MYC2 (green: G-Box, CACGTG; blue: PBE-Box, CATGTG; López-Vidriero et al. 2021) and BBX (pink: CAC[AGT]TG, red: G[AT]GAGAGA, orange: GAAA[AG][AT]GA, Li et al. 2024; grey: TGTGNNNATG, Tiwari et al. 2010) binding motifs in the promoter of *SlBBX17* and *SlMYC2*, respectively. Numbers indicate nucleotide positions upstream of the ATG. Transactivation assay of *pSlBBX17* by SlMYC2 and/or SlMED25; and of *pSlMYC2* by SlBBX17 in *N. tabacum* BY-2 protoplast cells. The promoter::fLUC and 35S::effector constructs are shown. FIREFLY LUCIFERASE (fLUC) activity is expressed as fLUC/RENILLA LUCIFERASE (rLUC) activity ratio relative to the negative control. Values are means ± SE (*n* = 6). Different letters denote statistically significant differences among samples (*P* < 0.05). **E and F** Yeast two-hybrid interactions between SlBBX17 and JA-mediated signaling proteins **(E)** and between SlMYC2 and SlJAZ4 or SlJAZ7 **(F)**. *AD* fusion to the activation domain; *BD* fusion to the binding domain; *EMPTY* autoactivation control; *-LT* positive control in non-selective medium without leucine and tryptophan; *-LTH* selective medium without leucine, tryptophan and histidine. The black boxes show two individual colonies at 10- (above) and 100-fold (below) dilutions

The aforementioned analyses highlight *SlBBX17* as the only *SlBBX* gene whose transcription is highly induced by JA in an SlMYC1/SlMYC2- and SlCOI1-dependent manner, suggesting that SlBBX17 may function as a regulator of the JA-mediated defense response. *SlBBX17* (Solyc07g052620) belongs to the Structural Group V of the BBX family and encodes a 130-amino acid protein with a single BBX domain (Fig. 3A-B, Supplementary Figure S7). In agreement with the prediction of a nuclear localization signal (Fig. 3B), the co-localization of the SlBBX17-GFP fusion protein with the DAPI nuclear marker, demonstrated the nuclear targeting of SlBBX17 (Fig. 3C). Consistent with the SlMYC2/SlMYC1-dependent expression of *SlBBX17*, we identified three SlMYC2-binding motifs within the *SlBBX17* promoter. Furthermore, transactivation assays in tobacco protoplasts demonstrated that, although SlMYC2 and SlMED25 individually enhanced *SlBBX17* transcription, their co-expression resulted in an approximately eightfold increase in *SlBBX17* promoter activity (Fig. 3D). Moreover, the *SlMYC2* promoter contains BBX-binding motifs, and SlBBX17 increases *SlMYC2* transcription by approximately 2.5-fold (Fig. 3D). These results reveal that *SlBBX17* is a direct target of SlMYC2 and suggest a potential regulatory feedback relationship, as SlBBX17 can directly induce *SlMYC2* promoter activity.

Given the importance of protein interaction for JA signaling, we investigated whether SlBBX17 physically interacts with core JA signal transduction factors, such as SlJAZs, SlMED25 and SlMYC2. From the tested proteins, only SlJAZ4 and SlJAZ7 interacted with SlBBX17 (Fig. 3E; Supplementary Figure S8). Similar to *SlBBX17*, *SlJAZ4* and *SlJAZ7* were strongly upregulated by JA in an SlCOI1-dependent manner and, at least initially, were also dependent on SlMYC1/SlMYC2 (Fig. 2). Interestingly, both SlJAZs interact with SlMYC2 (Fig. 3F). These results indicate that SlBBX17 is a JA-induced JAZ-interacting protein, belonging to the same transcriptional module as SlJAZ4 and SlJAZ7.

**Fig. 4:**
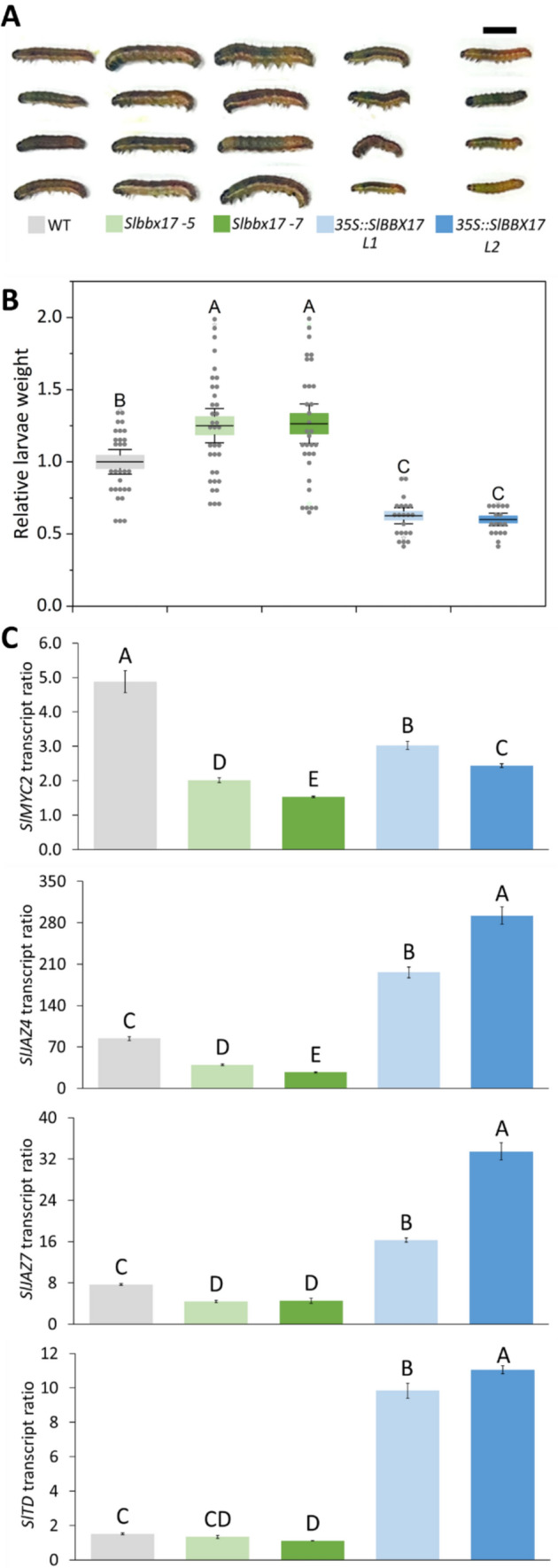
SlBBX17 regulates herbivory resistance and JA signaling. Representative images **(A)** and relative larvae weight **(B)** of *Spodoptera frugiperda* neonate larvae 5 days after being reared on leaves from each genotype. Values represent mean ± SE relative to the WT genotype from at least 26 biological replicates. Different letters denote statistically significant differences among samples (*P* < 0.05). Scale bar = 0.5 cm. **C** Transcript ratio (wounded/control) of *SlMYC2*, the SlBBX17-interactors *SlJAZ4* and *SlJAZ7*, and the wounding/herbivory marker *SlTD* in WT, *Slbbx17*, and *35S::SlBBX17* leaves 1 h after mechanical wounding. Values represent mean ± S.E of at least three biological replicates normalized against the control sample from the respective genotype. Different letters denote statistically significant differences among samples (*P* < 0.05). The relative transcript values are detailed in Supplementary Table S6

To assess the functional relevance of SlBBX17 in the context of plant defense, we generated two CRISPR/Cas9-mediated *Slbbx17*-knockout genotypes, harboring distinct null alleles with a premature stop codon in the encoded proteins (Supplementary Figure S1), and two constitutive *35S::SlBBX17-*overexpressing lines with an up to 40- and 100-fold higher *SlBBX17* transcript accumulation than WT plants (Supplementary Figure S2). Plants of these four genotypes were challenged with the herbivorous caterpillar *Spodoptera frugiperda* (Fig. 4A–B). Larvae feeding on leaves from *Slbbx17* plants attained a greater weight than those feeding on WT leaves, whereas larvae feeding on leaves of *35S::SlBBX17* plants showed a reduced weight gain. These results provide evidence that SlBBX17 positively regulates the herbivory defense response.

Finally, we evaluated the JA molecular signaling framework in the *SlBBX17*-deficient and -overexpressing genotypes by transcriptionally profiling *SlMYC2*, the SlBBX17-interactors *SlJAZ4* and *SlJAZ7*, and the wounding/herbivory marker *THREONINE DEAMINASE* (*SlTD*; Chen et al. 2005; Du et al. 2017) in response to mechanical wounding simulating herbivory (Fig. 4C, Supplementary Table S6). The lesion upregulated *SlMYC2* expression across all genotypes, albeit to varying degrees. The highest increase was observed in WT leaves (~ 4.8-fold), whereas *SlBBX17-*overexpressing and -deficient genotypes exhibited more moderate increases of approximately ~ 2.7-fold and ~ 1.7-fold, respectively. Both *SlJAZ* genes and *SlTD* exhibited similar transcriptional profiles: compared with WT plants, wounding-induced transcript accumulation was markedly higher in leaves from *35S::SlBBX17* plants and lower in those from *Slbbx17* mutants (Fig. 4C).

Overall, the transcriptional profile of *Slbbx17*-knockout plants indicated an attenuated response to wounding, consistent with their increased susceptibility to herbivory. In contrast, data from *35S::SlBBX17*-overexpressing genotypes pointed to a more complex regulatory scenario. Although the elevated expression of *SlJAZ*s and *SlTD* suggests an enhanced SlMYC2 activity, consistent with the observed resistance phenotype, the reduced expression of *SlMYC2* itself may reflect negative feedback mechanisms that downregulate this gene under conditions of heightened defense activation.

## Discussion

JA signaling is essential for plants to respond to various environmental stimuli, and finely regulates several plant metabolic and developmental processes (Goossens et al. 2016; Li et al. 2021; Nguyen et al. 2022). In this context, JAZ proteins serve as key regulators of the JA response, controlling the activity of MYC2, the master JA-related TF, and limiting its action when JA-Ile is present in the cell (Chini et al. 2016; Howe et al. 2018).

As additional JAZ interactors were identified, the roles of JAZ proteins in several physiological processes beyond the defense response became increasingly evident (Chini et al. 2016; Howe et al. 2018). In parallel, the multifaceted functions of BBX TFs have also been increasingly recognized (Lira et al. 2026). However, interactions between BBXs and JA signaling and/or JAZ proteins remain poorly characterized.

Here, we revisited the TIFY/JAZ protein family encoded in the tomato genome. While the identified proteins found were consistent with those previously reported (Chini et al. 2017), several nuances emerged. First, the JAZ clade was clearly subdivided into three distinct subgroups. Notably, the JAZ-I subgroup encompassed sequences with distinct characteristics, including two SlJAZs in which we observed amino acid residues under positive selection. Second, regarding the domain topology, the ZIM and ZML proteins were previously described as containing the TIFY, GATA, and CCT domains (Bai et al. 2011). Besides those, our analysis revealed the presence of a modified Jas domain also in the PPD proteins, where the degron and PY-NLS motifs were seemingly lost, while the core residues of the Jas motif remained mostly intact. Finally, we identified distinct sequences for the core motifs within the TIFY and Jas domains for each JAZ subgroup. Overall, the data obtained here provide a more comprehensive overview of the TIFY/JAZ protein family, enhancing our understanding of its domain topology and shedding light on variations in conserved motifs across different clades. Now, the impact of these divergences on protein functionality remains to be addressed.

Then, we analyzed the transcriptional profiles of *SlTIFY/JAZ* and *SlBBX* families in response to sustained MeJA exposure and the rapid transient response triggered after herbivory simulated by mechanical wounding. This combined analysis revealed three distinct expression patterns: genes with a strong upregulation, moderate upregulation or strong downregulation, particularly during the early response to JA. The transcriptional modulation of these gene families in response to JA has been previously reported. Chini et al. (2017) examined the expression of eight *SlJAZ* genes in the first leaf of hydroponically grown tomato plants and found that four genes were downregulated and four were upregulated in response to JA treatment. Regarding *SlBBX*s, Chu et al. (2016) assessed their modulation by JA in whole shoots of 1-month-old hydroponically grown tomato plants without observing any clear transcriptional pattern. The discrepancies between these results and those obtained here may be due to differences in growth conditions, organs sampled, and/or plant age.

Among the *SlBBX*s, *SlBBX17* stands out as the only gene clustering within the strongly JA-responsive group. Notably, among the few *SlBBX*s upregulated in response to herbivory by larvae of the leaf miner *Tuta absoluta* and the chewing lepidopterans *S. exigua* and *Manduca sexta*, *SlBBX17* is the one showing the highest increased expression level (Roumani et al. 2022; Lidoy et al. 2025). Consistent with the RNA-seq data obtained by Du et al. (2017), our analysis of *Slmyc1/Slmyc2* mutant hairy roots revealed that the JA-mediated upregulation of *SlBBX17* depends on SlMYC1 and/or SlMYC2. Furthermore, our transactivation assay confirmed that SlMYC2 directly activates the *SlBBX17* promoter, an effect that is enhanced in the presence of SlMED25. Interestingly, SlBBX17 can also induce the activity of *SlMYC2* promoter, suggesting that the SlMYC2-SlBBX17 hub may form a positive feedback loop that is triggered during early stages of JA signaling.

Consistent with this hypothesis, our data from the knockout and overexpressing genotypes show that SlBBX17 modulates the herbivory response: *Slbbx17* mutants are more susceptible to *S. frugiperda* larval feeding, whereas the *SlBBX17-*overexpressing lines display enhanced resistance. When the transcriptional responsiveness of *SlJAZ4*, *SlJAZ7*, *SlTD*, and *SlMYC2* to mechanical wounding was evaluated, clear patterns emerged. For all four analyzed genes, responsiveness was attenuated in *Slbbx17* leaves compared to the WT, reinforcing that SlBBX17 is involved in wounding-induced and JA-modulated signaling. In leaves from plants constitutively overexpressing *SlBBX17*, the responsiveness of *SlJAZ4*, *SlJAZ7*, and *SlTD* was enhanced, whereas that of *SlMYC2* was diminished. This reduction may appear contradictory given the herbivory-resistant phenotype. However, although wounding and herbivory trigger converging effectors, these stimuli are not fully equivalent. Herbivory involves not only mechanical injury but also the secretion of chemical elicitors, triggering a much more complex molecular and biochemical defense response (Walling 2000). Moreover, similar seemingly contradictory molecular responses have been reported following *MYC2* overexpression. Tomato plants overexpressing *SlMYC2* showed a reduced expression of JA-responsive marker genes after wounding. This reduction resulted from the upregulation of *MYC2-TARGETED BHLH* (*SlMTB*) genes, whose encoded proteins interact with SlMYC2 and constrain its activity (Liu et al. 2019). Similarly, constitutive overexpression of *AtMYC2* in *A. thaliana* leads to reduced transcript levels of endogenous *AtMYC2* (Dombrecht et al. 2007). Therefore, overexpression of high-hierarchy TFs may activate compensatory regulatory mechanisms that attenuate pathway activation, even when overall defense phenotypes are enhanced.

**Fig. 5:**
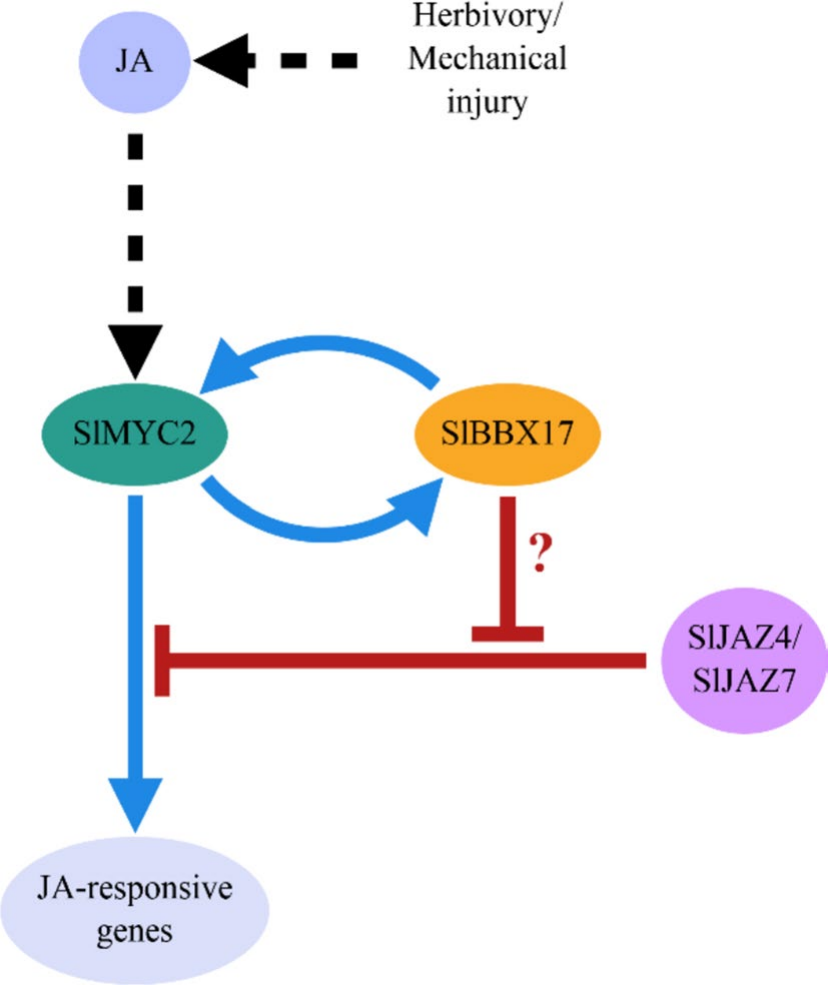
SlBBX17 positively modulates tomato defense. Following mechanical wounding that triggers JA accumulation, SlMYC2 is activated, inducing the expression of *SlBBX17* and other JA-responsive genes. In turn, SlBBX17 amplifies SlMYC2 activity by two mechanisms, directly upregulating *SlMYC2* expression, and by physical interaction with SlJAZ4 and SlJAZ7, alleviating their repression over SlMYC2 activity. Black dashed lines indicate that intermediary steps were omitted. Red and blue colors indicate protein–protein and transcriptional interactions, respectively. Lines ending with arrowheads and blunt-ends indicate positive and negative interaction, respectively. The question mark indicates the hypothesis that SlBBX17 attenuates SlJAZ-mediated repression of SlMYC2 activity

We also found that SlBBX17 interacts with SlJAZ4 and SlJAZ7. Notably, SlJAZ4 was recently identified as a negative regulator of tomato leaf resistance to herbivory (Wu et al. 2024), while SlJAZ7 dampens the JA response by competing with SlMED25 for the binding to SlMYC2, thereby reducing the activity of the latter (Liu et al. 2019). Accordingly, one can hypothesize that, following mechanical wounding, SlMYC2 induces *SlBBX17* expression, and its encoded protein might enhance SlMYC2 activity in two ways: by directly upregulating *SlMYC2* expression, and by physically interacting with SlJAZ4 and SlJAZ7, a possible mechanism that alleviates their repression over SlMYC2 activity (Fig. 5).

In conclusion, our results identify *SlBBX17* as a JA-responsive gene that acts as a positive modulator of JA signaling, fine-tuning plant defense responses against herbivores. These results highlight BBX TFs as components of the JA signaling network, expanding the current understanding of the regulatory architecture underlying plant stress responses.

## Supplementary Information

Below is the link to the electronic supplementary material.

Supplementary file1 (DOCX 1469 KB)

Supplementary file2 (XLSX 40 KB)

## Data Availability

All data supporting the findings of this study are available within the paper and its Supplementary Information.
