# Supplementary material for: The transcriptional dynamics of TIFY/JAZ and BBX families in jasmonate signaling reveal SlBBX17 as a positive regulator of tomato defense
Source: Plant Cell Rep. 2026 Jul 4;45(7):217. doi: 10.1007/s00299-026-03893-8 (PMC13333002; doi:10.1007/s00299-026-03893-8)
Supplement: Supplementary file 1 — Supplementary file1 (DOCX 1469 KB) [file 299_2026_3893_MOESM1_ESM.docx]

**Supplementary Figures**

**Manuscript:** The transcriptional dynamics of TIFY/JAZ and BBX families in jasmonate signaling reveal SlBBX17 as a positive regulator of plant defense in tomato

**Journal name:** Plant Cell Reports

**Authors:** Bruno Silvestre Lira^1^, Letícia Guimarães Barbosa^1^, Marcelo Lattarulo Campos^2^, Juliene Moreira^1^, Gabriel Ponciano^1^, Raquel Tsu Ay Wu^1^, Lumi Shiose^1^, Nikolaos Ntelkis^3,4^, Nathalia de Setta^5^, Alain Goossens^3,4,6^, Luciano Freschi^1^, Magdalena Rossi^1‡^.

**Affiliations:**

^1^ Departamento de Botânica, Instituto de Biociências, Universidade de São Paulo, Rua do Matão 277, 05508-090, São Paulo, Brasil.

^2^ Departamento de Botânica e Ecologia, Instituto de Biociências, Universidade Federal de Mato Grosso, Av. Fernando Corrêa da Costa 2367, 78060-900, Mato Grosso, Brasil.

^3^ Department of Plant Biotechnology and Bioinformatics, Ghent University, Technologiepark-Zwijnaarde 71, Ghent, Belgium.

^4^ Center for Plant Systems Biology, VIB, Technologiepark-Zwijnaarde 71, Ghent, Belgium.

^5^ Centro de Ciências Naturais e Humanas, Universidade Federal do ABC, Alameda da Universidade s/n, 09606-405, São Bernardo do Campo, Brasil.

^6^ Department of Botany and Zoology, Stellenbosch University, Stellenbosch, 7600, South Africa.

‡Corresponding author.

**Corresponding author e-mail:** mmrossi@usp.br


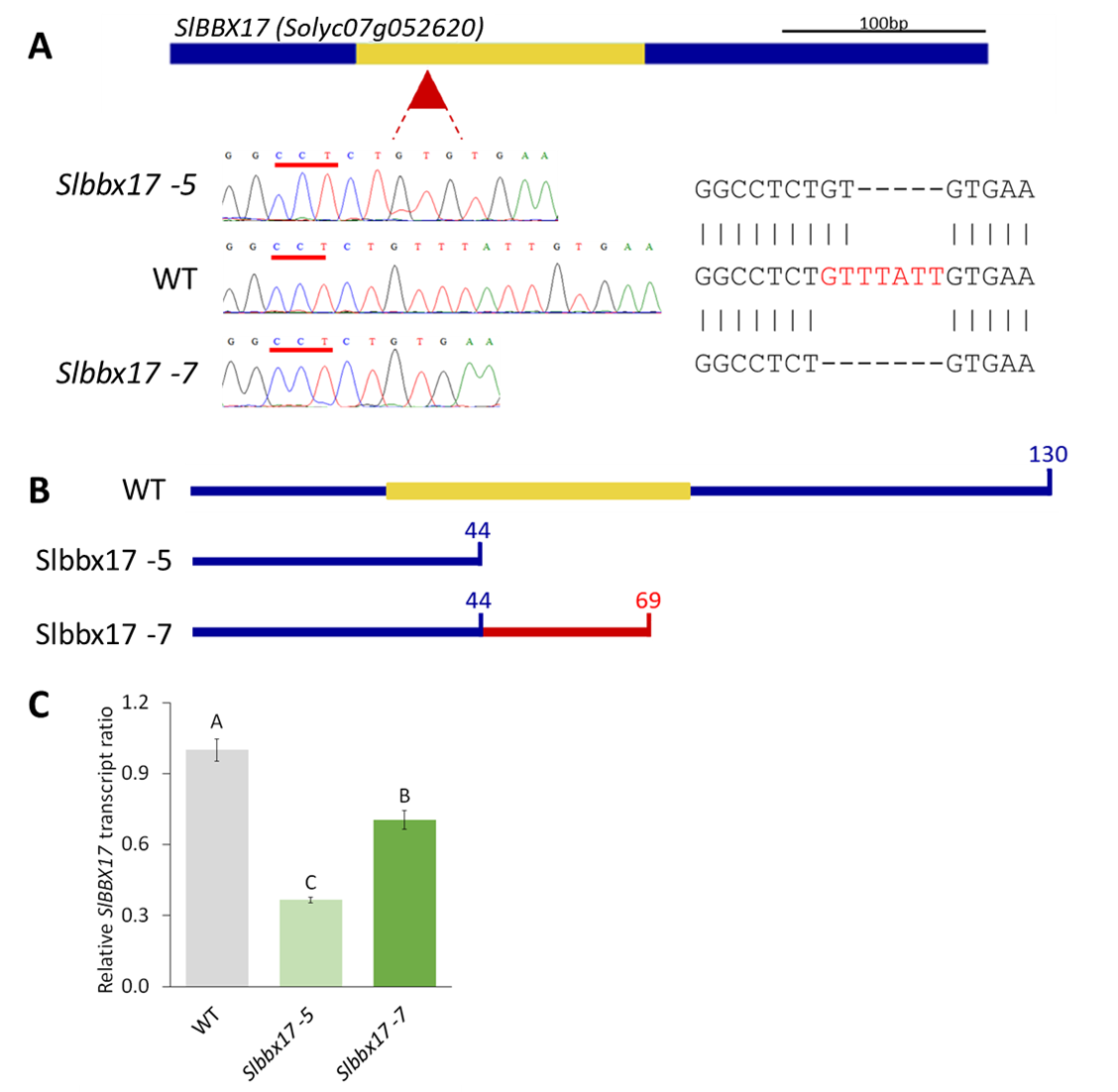


**Supplementary Figure S1. Characterization of *Slbbx17* edited alleles. (A)** Genomic structure of the wild type (WT) and edited knockout *Slbbx17* alleles with highlight of the edited region. The edited alleles were name following the number of deleted bases. Dark blue boxes: coding regions. Yellow box: B-BOX domain coding sequence. Red arrowhead: protospacer adjacent motif (PAM) downstream of the Cas9 target sequence. This gene does not contain introns. The sequence chromatograph and the alignment between WT and mutant alleles is shown. Underlined bases highlight the PAM sequence. Red bases in the alignments indicate the deletion in *Slbbx17* knockout alleles. **(B)** Schematic representation of the protein encoded by the WT and *Slbbx17-5* and *Slbbx17-7* alleles. Dark blue lines: regions without known domain. Yellow box: B-BOX domain. Red lines: amino acids encoded by the mutant allele that differ from the WT. Numbers indicate the protein length. **(C)** Relative transcript ratio of *SlBBX17* in leaves of 40-day-old WT and *Slbbx17* mutants. Values represent mean ± S.E of at least three biological replicates normalized against the WT sample. Different letters denote statistically significant differences among samples (*P* < 0.05).


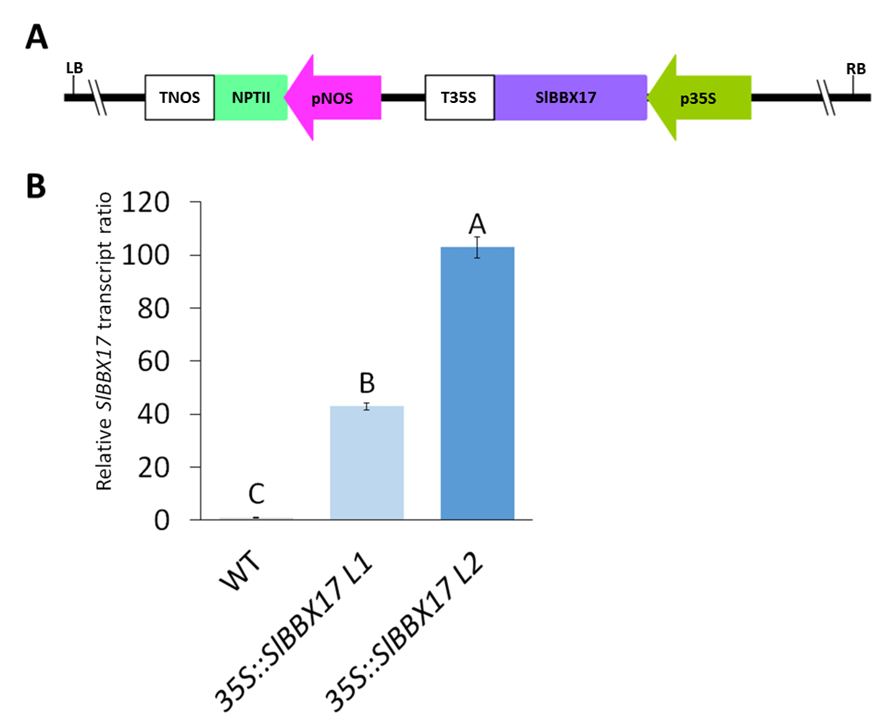


**Supplementary Figure S2. Characterization of *SlBBX17* overexpressing lines. (A)** Schematic representation of the T-DNA of the SlBBX17-pK7WG2D,1 construct designed for the generation of the *35S::SlBBX17* transgenic lines. RB and LB: left and right borders for *Agrobacterium tumefaciens*-mediated transformation. p35S: CaMV 35S promoter. *SlBBX17*: *SlBBX17* coding sequence. T35S: CaMV 35S terminator. pNOS: *NOPALINE SYNTHASE* promoter. NPTII: *NEOMYCIN PHOSPHOTRANSFERASE II* coding sequence. TNOS: *NOPALINE SYNTHASE* terminator. **(B)** Relative transcript ratio of *SlBBX17* in leaves of 40-day-old WT, *35S::SlBBX17-L1,* and *35S::SlBBX17-L2*. Values represent mean ± S.E of at least three biological replicates normalized against the WT sample. Different letters denote statistically significant differences among samples (*P* < 0.05).


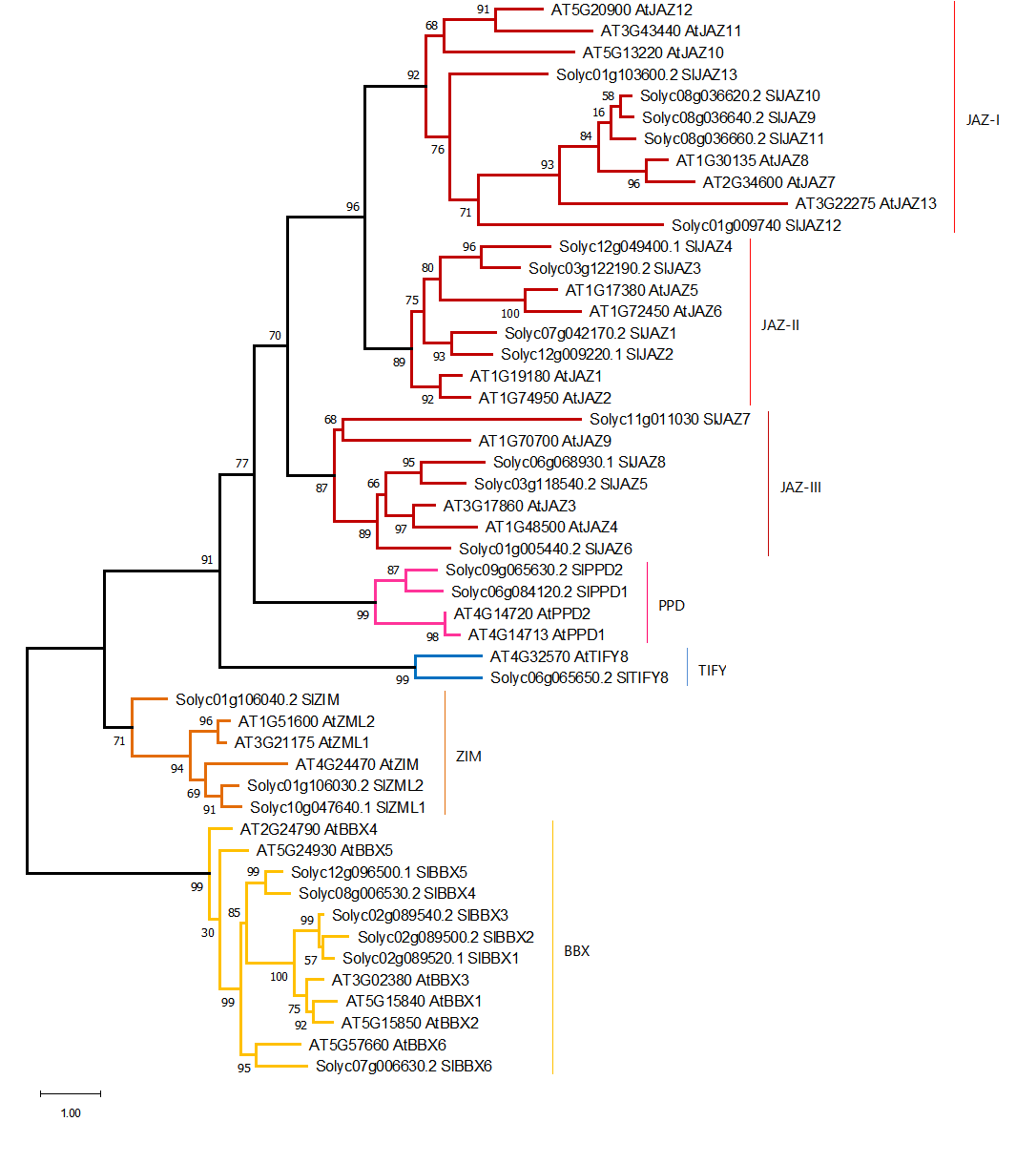


**Supplementary Figure S3. Phylogenetic analysis of *A. thaliana* and *S. lycopersicum* TIFY/JAZ proteins.** Phylogenetic reconstruction obtained from the alignment of *A. thaliana* and *S. lycopersicum* TIFY/JAZ proteins. Clusters were named according to the sequences contained within each group. The BBX sequences were used as outgroup for this analysis. Terminals are named with the locus ID (AT: *A. thaliana*; Solyc: tomato) and protein name. Sequences used in this reconstruction are listed in Supplementary Table S1.


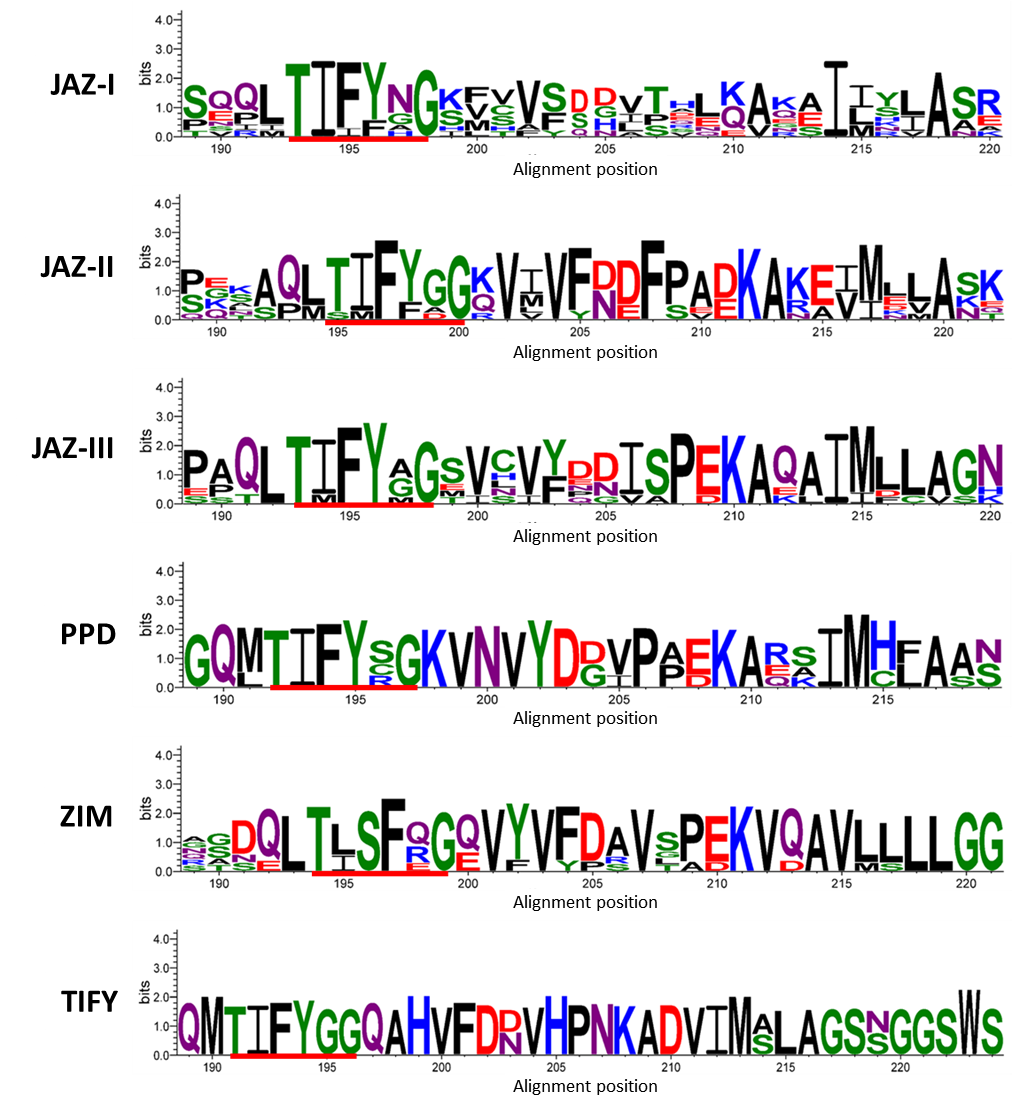


**Supplementary Figure S4. Conservation of the TIFY domain across TIFY/JAZ groups.** Graphical logo representation of the conservation of the TIFY domain (PF06200) in the TIFY/JAZ groups. The domain core motif is highlighted by a red line. For this analysis, each group was independently aligned. Representation was made with WebLogo 3 (Crooks et al. 2004, https://weblogo.threeplusone.com/).


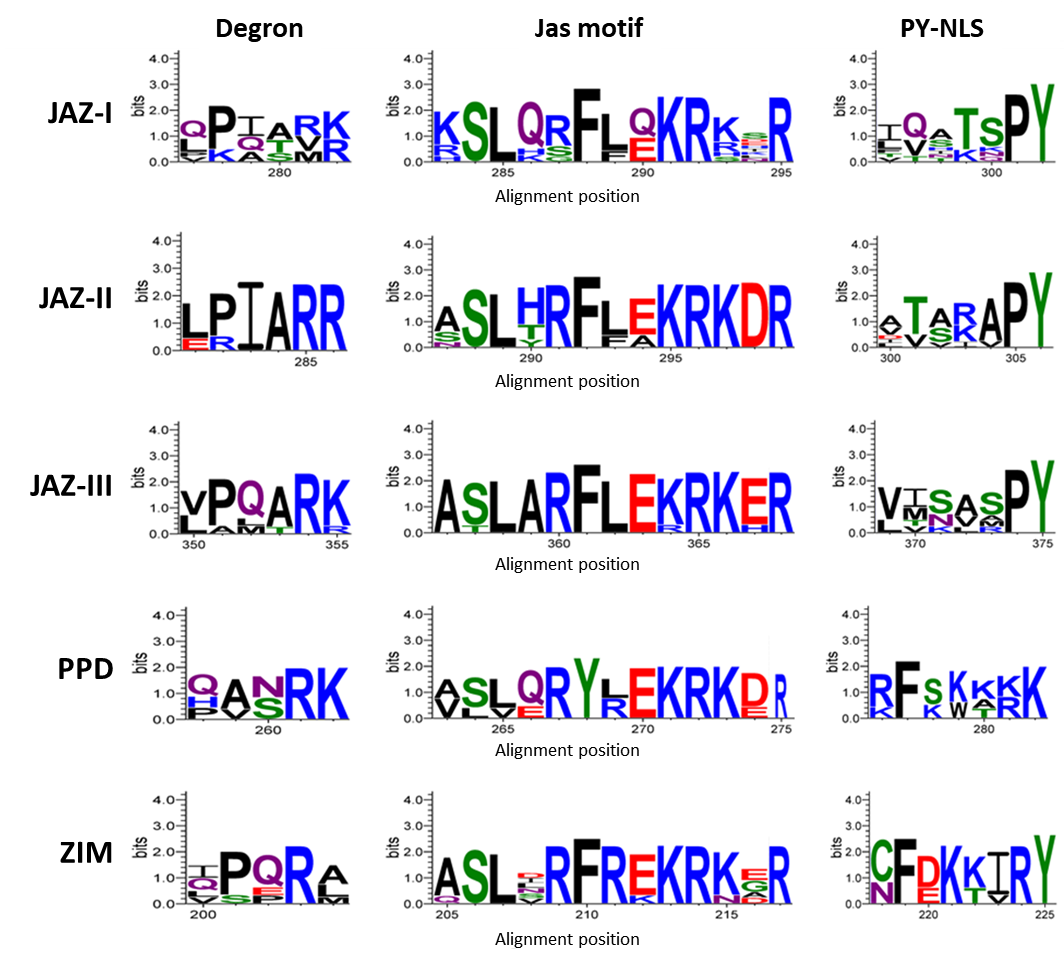


**Supplementary Figure S5. Conservation of the Jas domain across TIFY/JAZ groups.** Graphical logo representation of the conservation of the three subdivisions of the Jas domain (PF09425) in the TIFY/JAZ groups. For this analysis, each group was independently aligned. The Jas domain could not be identified in the proteins of the TIFY8 group; thus, this group was not represented. Representation was made with WebLogo 3 (Crooks et al., 2004; https://weblogo.threeplusone.com/).

**Supplementary Figure S6. Phylogenetic analysis of JAZ proteins from *Solanum* species.** Phylogenetic reconstruction obtained from the alignment of tomato *(Solanum lycopersicum*), potato (*Solanum tuberosum*)*,* and *Solanum pennellii* JAZ proteins. Clusters were named according to the distribution of tomato *JAZ* sequences following nomenclature of Figure 1. Terminals are named with the locus ID (Solyc: tomato; PGS: potato; Sopen: *Solanum pennellii*) and, for tomato, locus name. Sequences used in this reconstruction are listed in Supplementary Table 1.

**Supplementary Figure S7. Phylogenetic analysis of SlBBX proteins.** Phylogenetic reconstruction obtained from the alignment of tomato *(Solanum lycopersicum*) SlBBX. Clusters were named following the domain topology described in Lira et al. (2026). Terminals are named with the locus ID and protein name. Sequences used in this reconstruction are listed in Supplementary Table 1.


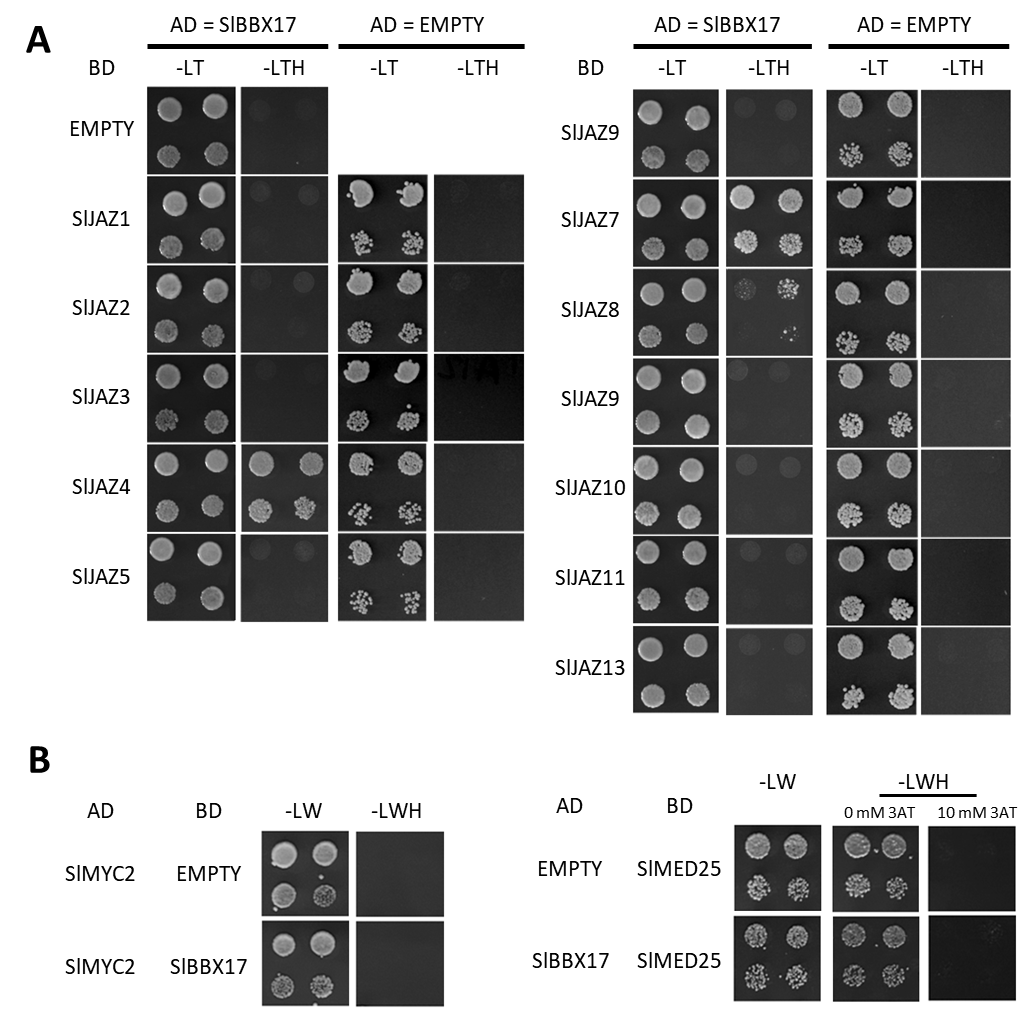


**Supplementary Figure S8. Physical interaction between SlBBX17 and JA-signaling proteins.** Yeast two-hybrid interactions between SlBBX17 and SlJAZs **(A)** and between SlBBX17 and SlMYC2 or SlMED25 **(B)**. AD: fusion to the activation domain; BD: fusion to the binding domain; EMPTY: autoactivation control; -LW: positive control in non-selective medium without leucine and tryptophan; -LWH, selective medium without leucine, tryptophan and histidine. 3AT: 3-aminotriazole for autoactivation inhibition. Black boxes show two individual colonies at 10- (above) and 100-fold (below) dilutions.
